# The role of schools in children and young people’s self-harm and suicide: systematic review and meta-ethnography of qualitative research

**DOI:** 10.1186/s12889-016-3065-2

**Published:** 2016-05-14

**Authors:** Rhiannon Evans, Chloe Hurrell

**Affiliations:** DECIPHer, School of Social Sciences, Cardiff University, 1-3 Museum Place, Cardiff, CF10 3BD UK

**Keywords:** Schools, Young people, Adolescent health, Health behaviours, Self-harm

## Abstract

**Background:**

Evidence reports that schools influence children and young people’s health behaviours across a range of outcomes. However there remains limited understanding of the mechanisms through which institutional features may structure self-harm and suicide. This paper reports on a systematic review and meta-ethnography of qualitative research exploring how schools influence self-harm and suicide in students.

**Methods:**

Systematic searches were conducted of nineteen databases from inception to June 2015. English language, primary research studies, utilising any qualitative research design to report on the influence of primary or secondary educational settings (or international equivalents) on children and young people’s self-harm and suicide were included. Two reviewers independently appraised studies against the inclusion criteria, assessed quality, and abstracted data. Data synthesis was conducted in adherence with Noblit and Hare’s meta-ethnographic approach. Of 6744 unique articles identified, six articles reporting on five studies were included in the meta-ethnography.

**Results:**

Five meta-themes emerged from the studies. First, self-harm is often rendered invisible within educational settings, meaning it is not prioritised within the curriculum despite students’ expressed need. Second, where self-harm transgresses institutional rules it may be treated as ‘bad behaviour’, meaning adequate support is denied. Third, schools’ informal management strategy of escalating incidents of self-harm to external ‘experts’ serves to contribute to non-help seeking behaviour amongst students who desire confidential support from teachers. Fourth, anxiety and stress associated with school performance may escalate self-harm and suicide. Fifth, bullying within the school context can contribute to self-harm, whilst some young people may engage in these practices as initiation into a social group.

**Conclusions:**

Schools may influence children and young people’s self-harm, although evidence of their impact on suicide remains limited. Prevention and intervention needs to acknowledge and accommodate these institutional-level factors. Studies included in this review are limited by their lack of conceptual richness, restricting the process of interpretative synthesis. Further qualitative research should focus on the continued development of theoretical and empirical insight into the relationship between institutional features and students’ self-harm and suicide.

## Background

Suicide amongst children and young people is a major public health concern [1]. Despite evidence of the routine underestimation of prevalence in this population [2], it remains the second lead cause of death in 15–29 year olds [1]. Self-harm is definitionally contentious, with some researchers differentiating between non-suicidal self-injury [NSSI] and acts that have an associated suicidal intent [3–5]. However, both behaviours share a number of risk factors [6], suggesting they be conceived as being along the same continuum [7]. Self-harm remains a risk factor for suicidal ideation [8] and completed suicide [9, 10]. Prevalence amongst adolescents ranges from 6.9 to 18.8 % in the UK [8, 11–13]. Suicidal ideation has been estimated at 15.8 % [8] and suicide attempt at 9.2 % [14]. Approximately a third of adolescent ideators go on to make an attempt on life [15], and prior suicide attempt if the most significant risk factor for suicide [1].

Multifarious settings have been implicated as sites for prevention, with schools offering some of the most extensive intervention opportunities [16–19]. Whilst the effectiveness of therapeutic interventions for non-suicidal self-harm has been demonstrated, including dialectical behaviour therapy, cognitive-behavioural therapy, and mentalization-based theory [20], there remains limited evidence for school-based assessment and treatment [21]. Tools to support school professionals are increasingly being made available however, including Self-injury Outreach and Support (SiOS). Prevention of suicide attempt and completed suicide has a stronger evidence-base, with intervention focusing on: awareness and education curricula; screening; gatekeeper training; skills training; and peer leadership [22]. Although evaluation has been hampered by methodological limitations [18], numerous interventions have demonstrated clear effectiveness [23], including the SOS suicide prevention program [24] and the Good Behaviour Game [25]. The Saving and Empowering Young Lives in Europe (SEYLE) study’s cluster-randomised controlled trial offers some of the most scientifically robust evidence, reporting that that the Youth Aware of Mental Health Programme was effective in reducing severe suicidal ideation by 50 % and incidents of suicide attempts by 55 % at 12 month follow-up [26].

Despite increased focused on school-based intervention, there remains a dearth of research exploring the role played by institutional features (both social and physical) in children and young people’s self-harm and suicide. Such examination is of vital importance for three key reasons. Firstly, school-level influences may serve as independent risk factors for self-harm and suicide. Secondly, interventions are increasingly conceptualised as the interaction of causal mechanisms and context in the generation of outcomes [27, 28], otherwise known as the CMO configuration [29], and to understand the theory of change underpinning school-based interventions it is necessary to understand the influence of the context in question. Thirdly school-level influences may moderate or mediate the relationship between other predictor variables and self-harm and suicide, which may have important implications for the development of effective intervention.

Other substantive health areas, notably substance use, smoking, and teenage pregnancy, have been shown to be independently associated with institutional-level factors. Schools with higher academic attainment and attendance than would be anticipated given the social profile of students have reported reduced prevalence of these health behaviours [30–33]. Kidger et al. [34] offer one of the only longitudinal studies reporting on the effect of schools on self-harm. Analysis of the ALSPAC birth cohort found that self-harm at age 16 was associated with earlier perceptions of school, which included not getting on with or feeling accepted by others, not liking school or classwork, and feelings that teachers are not clear about behaviour or fail to consistently address misbehaviour.

Although such studies are instructive in highlighting the causal relationship between institutional features and health outcomes, the *mechanisms* through which schools’ social and physical environments impact upon health remains under-theorised. Through the exploration of the lived experience of schooling, qualitative research serves as an important complement to quantitative studies by providing insight into these complex processes, offering direction for future epidemiological testing and instruction for the development of theoretically-informed intervention. Jamal et al.’s [35] meta-ethnography explores the pathways through which schools may structure adolescents’ detrimental health behaviours. Posited theories include: young people’s identity work, including the need to adopt ‘tough’ identities that encompass high risk behaviours; the configuration of un-owned and unsupervised school spaces; the importance of positive relationships between students and school staff; and the need to escape school. Such theorisation of causal mechanisms has been largely elided within self-harm and suicide research. This paper seeks to address this gap by reporting on a systematic review and meta-ethnography of qualitative studies examining the processes through which institutional features impacts upon children and young people’s self-harm and suicide.

## Methods

Conduct of the meta-ethnography was informed by the work of Noblit and Hare [36] in addition to methodological reports [37–39] and worked examples [40–42]. Formal reporting guidance for meta-ethnography is currently in development [43], and in the absence of standardised reporting procedures the present study draws upon the PRIMSA [44, 45] and RAMESES [46] publication standards.

### Eligibility criteria

Studies were identified from database inception to June 2015. All qualitative research designs were included. Study settings encompassed primary or secondary education (and international equivalents) or alternative educational settings (e.g. Pupil Referral Units). Study participants were not restricted and could include reporting by any individual (e.g. students, teachers, or other educational professionals). Studies were required to report on the influence of educational settings on at least one of the following outcomes: self-harm (defined with or without suicidal intent); suicidal ideation; suicide attempt; completed suicide. Studies were limited to those published in the English language.

### Data sources and search strategy

Campbell et al.’s [37] guidance structured searching, and a sensitive strategy was developed in Ovid MEDLINE before being adapted to the search functions of each database. Substantive search terms were generated through consultation of relevant research. Methodological search terms were informed by technical guidance and worked examples of meta-ethnographies [37, 40, 41]. Nineteen electronic bibliographic databases were searched. These included: Applied Social Sciences Index and Abstracts (ASSI); British Education Index; The Campbell Library; CINAHL (the Cumulative Index to Nursing and Allied Health Literature); Cochrane Controlled Trials Database; Embase; ERIC (Education Resources Information Center); EPPI Centre DoPHER; HMIC (Health Management Information Consortium); Medline; Medline in Process; OpenGrey; Proquest Dissertations and Theses; PsycINFO (OVID); Social Care Online; Social Science Citation Index; Social Services Abstracts; Sociological Abstracts; Scopus. International experts were contacted for recommendations of relevant published and unpublished studies. Reference lists of included studies were scanned to identify additional publications.

### Study selection

**Fig. 1:**
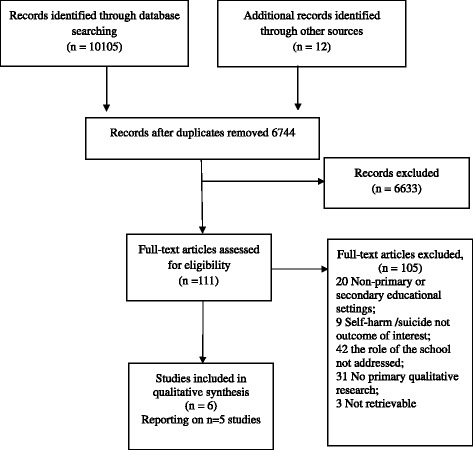
Flow Diagram of Study Retrieval. Flowchart of study retrieval adapted from the Preferred Reporting Items for Systematic Review and Meta-analyses (PRISMA) flow diagram. 6744 unique articles were retrieved. Six articles reporting on five studies met the inclusion criteria

Retrieved studies were exported into EndNote reference management package and duplicates were removed. Two review authors independently screened study titles and abstracts. Studies progressed to the next stage of screening if there were discrepancies between reviewers’ decisions. The full-texts of remaining studies were appraised against the inclusion criteria. Disagreement between reviewers was resolved through discussion. Reasons for exclusion at full-text are reported in Fig. 1.

### Data extraction

A standardised data extraction form was developed and was informed by Noyes & Lewin’s [47] *Supplementary Guidance for Inclusion of Qualitative Research in Cochrane Systematic Reviews of Interventions*. Extracted data included: context of the study; characteristics of study participants, sample size and sampling strategy; research methodology; researcher reflexivity; results, including details of the analytical frame and interpretation of data; initial observations of strengths and limitations. Two reviewers independently extracted data. Data abstraction did not form part of a linear review process, and in practice full-texts were routinely returned to during synthesis in order to re-contextualise data.

### Quality appraisal

Despite contestation over what constitutes high quality qualitative research [39], included studies were appraised using criteria adapted from Campbell et al.’s [37] technical report and the CASP critical appraisal checklist for qualitative research [48]. Appraisal items included: appropriateness of the methodology in addressing the research aim; strength of theoretical perspective; rigour of sampling and data collection; adequacy of data analysis; reflexivity and researcher bias; justification of data interpretation; transferability and relevance. In alignment with the CASP checklist studies were assigned a yes or no depending on whether they assessed a specified quality criterion and were marked as unclear if there was ambiguity or there was inadequate reporting. The appraisal tool was piloted and calibrated by the review team using a sub-sample of studies. Two reviewers independently appraised study quality, with disagreement being resolved through discussion. Studies were not excluded on the grounds of low quality.

### Analysis and synthesis processes

Study synthesis was informed by Noblit and Hare’s [36] methodological guidance. Comprising seven steps, the method can broadly be defined as drawing together findings from individual interpretive accounts to produce new interpretations and theoretical insights [39]. The first two phases pertain to the aforementioned formulation of the research question, searching and identification of relevant studies. The additional steps involve:

- *Phase 3: Reading the studies.*
  Studies were repeatedly read by the review team to record the context of the research and become acquainted with the concepts of interest. For example, it became apparent that vernacular and metaphors around visibility were latent in many studies, although had rarely been brought to conceptual fruition. Key themes drawn out during initial reading are presented in Table 1.

**Table 1:** Context, Design and Key Themes of Included Studies. Table presents overview of study characteristics and includes details on: country; school type; socio-demographic profile of school; study participants; sample size at both school and individual level; qualitative research method utilised; health outcome assessed; key themes and concepts to emerge from study data

Study	Country	School type	Profile of School	Participants	Sample size (schools; participants)	Research method	Health outcome	Key themes and concepts
Best (2006) [49]	UK	Secondary school	Not reported	Staff	Staff: *n* = 34	Interview	Self-harm	(1) Different levels of awareness across staff;
								(2) Range of staff’s interpretations of self-harm;
								(3) Panic and fear amongst staff during intervention;
								(4) Desire to relieve ‘the burden’ of intervening;
Coombes et al. 2013 [50]	UK	Secondary school	1 boys grammar school; 1 girls high school; 1 mixed sex grammar school; 2 mixed sex community colleges; Range of educational statements: 1–27; Range of students on CPR: 0–1; Range of male staff: 21 %–58 %	Students	Schools: *n* = 5	Focus group	Self-harm	(1) Omission of self-harm from the school curriculum;
					Students: *n* = Not reported;			(2) Different levels of understanding across students;
					Age:13–14 years;			
Mak (2011) [50]	Hong Kong	Secondary school	Not reported	Students	Schools: *n* = 3;	Interview	Suicide	(1) Pressure to be a high academic achiever;
					Students: *n* = 30;			
					Age 13–17 years;			
					Males = 7, Females = 23			
McAndrew & Warne (2014) [51]	UK	Secondary school	Not reported	Students	Students: *n* = 7;	Interview	Self-harm	(1) Bullying as a trigger factor;
								(2) Importance of supportive teachers young people can talk to;
					Age 13–17 years;			
								(4) Dismissive or disengaged staff;
					Females = 7			
								(3) Lack of information and support;
Simm et al. (2008) [52]	UK	Primary school	Not reported	Staff	Schools: *n* = 6	Interview with vignette	Self-harm	(1) Different levels of awareness across staff;
					Staff: *n* = 15			(2) Range of staff’s interpretations of self-harm
Simm et al. (2010) [53]	UK	Primary school	2 co-ed schools; one girls-only school	Staff	Schools: *n* = 6	Interview with vignette	Self-harm	(1) Different levels of awareness across staff;
								(2) Range of staff’s interpretations of self-harm
					Staff: *n* = 15			
								(3) Frustration amongst staff during intervention;
								(3) Omission of self-harm from the school curriculum;
								(4) Conflicting views on inclusion of self-harm in the curriculum
- *Phase 4: Determining how studies are related.*
  During data extraction the review team noted the key concepts, vernacular and metaphors that were used in each study and started to map their relationship across studies. This included discrepancies, tensions and consistencies in data. From here concepts were developed to serve as over-arching thematic descriptions of the data presented within and between individual studies (Fig. 2). Of note is that studies reported data from school staff or students. At this stage the distinction between sources were retained, and although the data were juxtaposed and understood in relation to each other, it was only during next stage that they were fully integrated.

**Fig. 2:**
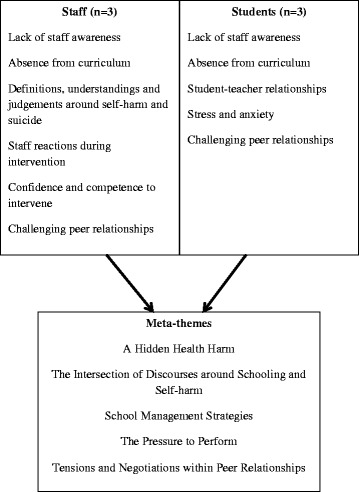
Overarching Staff and Student Themes and Meta-themes. Concepts, vernacular and metaphors were mapped and compared across study to develop over-arching thematic descriptions of the data presented within and between individual studies. These themes retained the distinction between staff and student data sources. Presented staff-level themes are: lack of staff awareness; absence from curriculum; definitions, understandings and judgements around self-harm and suicide; staff reactions during intervention; confidence and competence to intervene; and challenging peer relationships. Presented student-level themes are: lack of staff awareness; absence from curriculum; student-staff relationships; stress and anxiety; challenging peer relationships. Meta-themes were generated through the process of reciprocal translation in order to generate more rounded and nuanced themes that integrated both staff and student data. Presented meta-themes are: a hidden health harm; the intersection of discourses around schooling and self-harm; school management strategies; the pressure to perform; and tension and negotiations within peer relationships
- *Phase 5: Translating studies into one another.*
  This stage involved the generation of third-order ‘meta-themes’ [41]. Through the process of reciprocal translation concepts and metaphors were compared across studies (and thus across data sources) in order to generate rounded and nuanced themes that still retained the structural relationships of the concepts and metaphors presented within the original accounts. For example, the meta-theme of school management strategies compared the presentation of staff’s understanding and responses to self-harm disclosures across studies, whilst translating these findings into the student-level data that explored the experience of revealing self-injury to teachers.
- *Phase 6: Synthesising translations.*
  In response to the translations generated during Phase 5, a broader interpretative reading of the meta-themes was undertaken to develop an over-arching understanding of the role of schools in children and young people’s self-harm and suicide, with this interpretation retaining the key trope of ‘visibility’. This argument is presented in the discussion.
- *Phase 7: Expressing the synthesis.*
  Communication of the synthesis was deemed to be most appropriate in the written form due to an intended academic audience. In addition to discussion of the conceptual presentations by study authors, quotes from the participants of primary studies are included to ground the review in their lived experiences.

## Results

Searching of electronic bibliographic databases retrieved 10,105 studies. Consultation with experts identified a further twelve. After duplicate removal 6744 studies remained. Screening of title and abstract excluded 6633 studies, leaving 111 full texts for appraisal. There was initial disagreement on two studies, due to ambiguity over whether the setting was secondary or tertiary education, but the studies where subsequently excluded due to being conducted with further education students. Six articles reporting on five studies were included in the review [38, 39, 41–44]. The process of study identification, screening and selection is documented in Fig. 1.

Of the five reported studies, four were conducted in the UK [49–53] and one in Hong Kong [54]. Four of the studies were set in secondary schools [49–51, 54] and one in primary school [52, 53]. Two drew upon school staff as their research participants [49, 52, 53] and three focused on students’ lived experiences [50, 51, 54]. The two studies conducted with school staff utilised semi-structured interviews [49, 52, 53], with Simm et al. [52, 53] also including a vignette to prompt discussion. The vignette depicted a scenario of a boy banging his head, and participants were asked to consider their response, discuss their understanding of the head banging, and describe how the school might react. One study conducted with students carried out eight single-sex focus groups [50], one study employed semi-structured interviews [54], and the third study undertook narrative interviews with young people who had prior experience of self-harm and/or suicidal behaviour. Four of the studies addressed self-harm [49–53], with only one focusing on suicide [54]. The dominance of self-harm within the literature ensures that the following results are primarily focused on this outcome.

### A hidden health harm: The invisibility of self-harm within schools

Studies were suffused with the notion of self-harm as an invisible problem, and although participants acknowledged its escalating prevalence in some distal or abstracted reality, it was not necessarily observed or understood within their respective institutions and only the most severe acts were detected [49, 52]. Indeed, authors discussed behaviours that were hidden by students, unaware of by staff, and undefined by the curriculum:

*It’s like anorexics and bulimic behaviour; yes, I think people have always done that but we didn’t necessarily identify that. I mean certainly one of the teachers in my Department…*[]…*knows an adult who has psychiatric problems and she cuts a lot…*[but]…*it’s something that people keep to themselves. It’s not something people talk about…’*

(Int 8: Head of SEN, mixed comprehensive. Best [49]: 166)

Structural factors offer part explanation of why self-injury may be rendered invisible, with the conflicting and ever increasing demands placed on staff time allowing them to go unnoticed:

*Everybody’s busy, aren’t they…some people…oh they’ve got this to do, that to do,… they’d probably rush by and say ‘oh’, and not actually pick up on it.*

(Participant 7. Simm et al. [52]: 264)

In the absence of this behaviour being ‘seen’ within schools, it is not only evident why staff might underestimate the prevalence of self-harm [49, 53], it is also apparent how it fails to be elevated to priority status in schools and allocated resources [53]. Both staff and students reported the undervaluing or complete omission of self-harm from the mainstream curriculum [50, 53], despite young people’s expressed desire for further knowledge [50, 51]:

*Self-harm is attention seeking, but for some it’s because of depression…we’re not taught about it, we need to know…the school doesn’t want to admit it…cutting is attention seeking…it’s normality now.*

(FG82. Coombes et al. [50]: 229)

*There are posters all around school* (for smoking), *but then there’s nothing for counselling or anything like that. In my school, there are more people who actually self-harm than smoke or drink. Have an assembly about self-harming.*

(Lizzie. McAndrew & Warne [51]: 575)

Where discourses of shame and stigma abound around self-harm and suicide [51], institutional investment in their marginalisation may only serve to further perpetuate secrecy and reticence to seek help. Although staff were often keen to bring these behaviours to light, through the introduction of staff training that raised awareness [52], some feared their integration into the school curriculum due to the belief that ‘*talking about it would ‘put ideas in their head’ and encourage them to do it’* [53]. Moreover, as Best [49] maintains, invisibility could actually be accompanied by a desire not to be aware as open discussion could be blamed for a student self-harming.

### The intersection of discourses around schooling and self-harm: dealing with detection within context

Multifarious discourses structure understandings of self-harm, from empathetic interpretations of it being an expression of young people’s emotional pain to feelings of it being attention-seeking behaviour requiring censure [49, 53]. Within the school context these discourses are reified or mediated as they interact with the educational ethos that permeates the setting. In the first instance, some manifestations of self-harm are conceived as ‘bad behaviour’ where they transgress institutional norms and rules, leading to the denial of adequate support. Simm et al. [53] present the case of a teacher observing self-injury as disruptive and problematic, responding by sending the student out of the classroom and subsequently punishing them for being an annoyance. Indeed, self-harm was seen as a provocative display that was intended to detract from the learning process:

*I think most of its experimenting, but you probably get the odd child who, you know there’s a bit of peer pressure there to beat, I don’t know, showing off sort of thing.*

(Participant 15. Simm et al. [53]: 686)

Secondly, in settings that are enculturated with notions of progress and success, school staff may feel disempowered and lacking competence where students do not demonstrate consistent improvement in the reduction of self-harming behaviours following intervention [53]. Such feelings have the potential to contribute to a reticence to offer continued support, amidst a tendency to conceive behaviour maintenance rather than elimination as failure.

### School management strategies: Responding to staff competency and confidence

Although studies consistently reported a dearth of formalised strategies for managing self-harm, some practices had become entrenched. Derived from a need to seek ‘expertise’, these practices were often grounded in the fear, denial and panic expressed by school staff, with teachers being reported as being keen to refer the student on in the attempt to relieve the burden:

*It’s this panic. As soon as someone says “Oh, I’m doing this”, it’s like, I feel it’s some sort of panic: “Oh my God! What are we going to do? Oh my God!” you know?… And it’s all this: “Let’s quick, blah bah blah…”*

(Int 11: learning mentor, mixed comprehensive. Best [49]: 169)

*…*[T]* there are teachers who have got an awareness[-] but[…] think: “I’ve got to get rid of that. I don’t need to hold on to that right now. I just want somebody to help this young person”. And that’s a totally understandable human response.*

(Int 32: CAMHS staff. Best [49]: 170)

Such reactions had often led to an escalation approach. This entailed communicating the perceived problem through the institutional hierarchy to senior management, before making a referral to external ‘experts’ where necessary [44]. One student described this strategy as ‘Chinese whispers’ [39].

Whilst such procedures are understandable in the effort to secure the most appropriate support, harmful impacts were reported by students [49]. A number of individuals cited the importance of school staff as sources of help:

*Year Head and the Deputy Year Head, they’ve been very supportive, because you can go to them at any time and they’ll sit you down and let you talk to them.*

(Kim. McAndrew & Warne [51]: 574)

However, despite wanting to make a disclosure, students often encountered staff who appeared reluctant to engage in discussion:

*I never told them that I self-harmed. I did tell them that I was depressed and I’d had these suicidal thoughts, but she never said anything. She was like a nurse. If I had a broken, a leg I’d go straight to her for a bandage or whatever, but she never said anything.*

(Julie. McAndrew & Warne [42]: 575)

This disjuncture between school’s management strategies and students reporting may contribute to the latter not wanting to seek help, whilst serving to perpetuate the unseen nature of self-harm.

### The pressure to perform: teacher, parent and peer expectations of academic achievement

Anxiety and stress associated with school performance may encourage engagement in self-harm and suicide. Academic pressure was reported across both primary and secondary schools, where self-injury was a mechanism for coping or regaining control:

*I don’t think I would be very surprised to see a primary school child who started to self-harm purely and simply because the work was too difficult in school, or…because they were having some problem in school.*

(Participant 9. Simm et al. [53]: 685).

There may be a cultural dimension to the experience of academic pressure, with Mak et al. [54] citing a distinct social expectation to achieve a satisfactory school performance in Hong Kong, with this expectation being expressed by parents, teachers and peers:

*Young people feel pressure from potential poor academic achievement, social relationship, social interaction and bullies in school. They are suffering from examination anxiety because of the fear of poor grades and the fear of disappointing their parents, etc.*

(Participant 16. Mak et al. [54]: 47)

*I have suicidal ideation because they are much news about suicide initiated by poor academic performance. Many students committed suicide because* [they] *cannot accept their disappointing marks.*

(Participant 9. Mak et al. [54]: 47)

Although school staff were generally presented as being sympathetic, on occasion self-harm was perceived as an attempt to manipulate a situation, gain attention and distract from having to complete schoolwork [53]. Imputation of such negatively orientated explanations has the potential to diminish staff’s inclination to offer support, as it is not seen as signalling a need for assistance.

### Tensions and negotiation within peer relationships: Bullying and initiation within the school setting

Studies reported on the challenges of forming and sustaining relationships, with peer pressure and bullying frequently manifesting within the school context [51, 54]:

*It (being bullied) was bad in primary school, but it was not as bad as high school, but it was still bad. It’s really hard for me to stand up for myself…I didn’t really stand up for myself. I don’t think I have the confidence, so then I always used to keep it in’.*

(Tina. McAndrew & Warne [51]: 573)

Through the interaction with multiple other stressful life events, bullying could trigger or sustain engagement in self-harm:

*It was just all stress at once: stress from school and stress from people, friends being horrible people, and the family arguing.*

(Lizzie. McAndrew & Warne [51]: 573)

*The girl that carved…into her skin was…very unhappy with her relationships with her friends in school.*

(Participant 11. Simm et al. [53]: 685)

Such behaviour was not always conceived as a reaction to negative peer relations however, and could be exhibited in order to facilitate acceptance into a social group, essentially serving as an act of initiation [53].

Although such examples of self-harm may elide the role of school, in the sense that they are interpreted as a response to interpersonal relations between students, the fact that educational institutions are potentially passive sites where these relational negotiations and tensions play out suggests that they may bear some responsibility through inaction.

## Discussion

The present systematic review and meta-ethnography reveals that despite an emerging epidemiological body of research articulating the influence of schools on children and young people’s self-harm and suicide, these ideas have not yet been translated into conceptually rich, qualitative research. Such research is imperative in informing future epidemiological testing and developing theoretically informed interventions. Yet, the focus of existing empirical work centres on staff’s understanding and management of instances of self-harm, with only minimal consideration of the contribution of institutional features. Thus when located within socio-ecological models aimed at the theorisation of influences on health behaviours and outcomes [55], we must acknowledge empirical concentration within the interpersonal as opposed to the organisational domain. Nevertheless, through the interpretation of the meta-themes a ‘line of argument’ [40] may be formulated. We must recognise however that the extent of any new interpretation is limited by the majority of studies being conceptually ‘thin’ [38].

### Line of argument: the prevalence paradox

Schools recognise that self-harm and suicide is a problem, amidst fears that the pressures of modern society is contributing to a youth population with ever deteriorating well-being. However, the notion of ‘othering’ is inherent to discussion and debate, whereby schools focus on the classification of those who are different, whilst simultaneously distancing themselves from these ‘others’. Therefore, despite recognition of the phenomena of self-harm and suicide at the abstract, population–level, schools often view it as happening in other schools, amongst other students. This can lead to these behaviours being rendered invisible within their respective setting, with two key processes amplifying this invisibility. Firstly, self-harming behaviours are socially constructed and must be definitionally brought into being before they can be acknowledged. Often only the most severe forms of self-harm are defined as such by school staff, and thus many behaviours are rendered invisible [52]. Secondly, structural barriers, in terms of the time that staff can allocate to individual students, minimises opportunities for detection and disclosure.

The ‘unseen’ nature of these behaviours ensures that the prevalence of self-harm is significantly underestimated by staff [49, 53]. As a result, it is not prioritised, with the resourcing of prevention and intervention activities reflecting the perceived scale of the problem [53]. Self-harm remains missing from the curriculum despite suggestion that students desire exposure to information, and understand self-injury to be more prevalent than other health harms [50]. Equally, structures and supports systems to equip staff in prevention and intervention are rarely provided, with schools routinely escalating instances of harm through hierarchical structures in an effort to locate ‘expertise’. This sits in contrast to the needs of students, who value communication with staff about the issue and recognise the importance of being listened to [35]. Such sentiments indicate discontinuities with the broader suicidology literature around help seeking, where there is clear evidence that friends and families rather than teachers are the primary sources of interpersonal support [56, 57]. However, documented barriers to help-seeking suggest that there may not be inherent problems with seeking help from teachers but rather there may be a lack of intimacy within the relationship, fears of being deemed ‘attention-seeking’, or concerns around breaches of confidentiality [56, 57]. Data presented in this review suggest these barriers are likely present within schools, and in the absence of opportunities for staff and students to form meaningful relationships, secrecy and stigma may continue, which can inhibit help-seeking when trigger events such as peer conflict or academic pressure emerge.

Given the dearth of clear, positive and consistent approaches to the interpretation and management of self-harm, understandings of these behaviours are likely framed by the broader educational discourses that permeate the setting, which may resonate with the academically-orientated standards driven agenda. Where the trope of ‘bad behaviour’ is routinely used to construct and manage students’ identities, those engaging in self-harm or suicide-related behaviours may potentially be positioned as poorly behaved when alternative interpretative frameworks are unavailable. Punishment may ensue due to a perceived transgression of endorsed rules, serving to perpetuate shame. Equally, the juxtaposition of educational systems orientated to improvement and the non-linear self-harm journeys of young people may problematize the offering of support by schools, where staff feel frustrated or disempowered by students’ lack of clear ‘progress’ following provision of help.

### Limitations

The review is limited by the number and depth of available studies. Although Ring et al. [39] state that meta-ethnographies should work towards theoretical saturation rather than comprehensiveness, we are reticent to claim that saturation has been realised. Quality appraisal revealed a lack of conceptual richness. Only Simm et al. [52, 53] engaged in developing new theoretical insights through formulation of ‘the domino effect’ model, which foregrounded metaphors around visibility. Within this model, schools are understood as not being aware of self-harm because staff only have a limited conceptualisation of what constitutes self-harming behaviour (e.g. cutting), which prevents them from observing the full continuum of harmful behaviours that students engage in. Interpretative synthesis was limited as a result, with this study potentially being over-privileged in the new line of argument. However, as France et al. [38] maintain, the quality of studies are revealed by how much they contribute to the synthesis and as Simm et al. [52, 53] was the most conceptually comprehensive it is deserving of its privileged status. We have also progressed this work through the integration of both staff and student accounts, allowing for a more nuanced higher order explanation.

The range of qualitative data also restricts the potential scope and applicability of the concepts developed. Only one study addressed suicide [54], but the quality was poor and so the relevance of the review beyond self-harm should be treated with caution. Concentration of studies in Westernised counties, which may by characterised by some shared discourses around self-harm and suicide, potentially prevents the extension of findings to other cultural contexts where different interpretations prevail. However, despite some variation in settings, the consistency of themes across studies remained striking, suggesting some scope for generalizability.

Limitations are also inherent to the conduct and presentation of the meta-ethnography, which reflect broader conceptual and methodological debates [38]. Whilst a range of technical reports and worked examples have informed the review [40–42], the lack of consistency across the field has ensured that terminology, such as the ordering of concepts, are not clearly defined. The present review has sought to be explicit in regard to methodological conduct, but recognises that it may have contributed to this ambiguity. Forthcoming guidance by France et al. [43] may go some way in introducing standardisation to reporting procedures.

### Implications for future research

The present systematic review and meta-ethnography has highlighted the limited qualitative research theorising how schools’ institutional features structure self-harm, with a significant death of empirical work considering suicide. This may be unsurprising given broader concerns about the general absence of conceptual work within suicidology [58]. It is imperative to redress this lack, as qualitative methods are distinct in their capacity to offer insights into the complex, recursive and often unanticipated relationships between institutional-level influences and students’ health behaviours. Where public health increasingly presents ‘context’ as an inherent aspect of programme theory [27, 28], conduct of such research may encourage the development of better theoretically-informed, contextually responsive intervention. Such research might benefit from focusing further on the perspectives of young people, exploring both the positive and negative aspects of institutional-level features, including whether enhanced visibility may lead to the false interpretation of some behaviours as problematic, or increase surveillance to the extent that it causes anxiety and exacerbates the problem [49]. In addition to generating new theoretical insights, research might also contribute to the refinement of causal relationships that have been theorised or substantiated within the existing literature, notably around notions of school connectedness, relationships with peers and staff, and enjoyment of schoolwork [34, 59–61].

## Conclusions

Qualitative research highlights the mechanisms through which the institutional features of schools may impact upon student self-harm. There is limited indication of a role in suicide. Evidence suggests that organisational practices serve to render self-harm invisible, which may inhibit the provision of comprehensive preventative or intervention approaches. Further qualitative research is required to continue the theorisation of the role of educational institutions in explaining children and young people’s self-harming and suicidal behaviours.

### Ethics approval and consent to participate

Not applicable.

### Consent for publication

Not applicable.

### Availability of data and materials

Original materials are attached as Appendices 1, 2, and 3.

## Appendix 1: Search strategy

**Table 2:** Initial detailed Medline Search 1946 to June Week 2 2015 (Includes EMBASE 1947-Present, Ovid MEDLINE(R) In-Process & Other Non-Indexed Citations June 23, 2015, PsycINFO1806 to June Week 3 2015, HMIC (Health Management Information Consortium)

#	Search history	Results
1.	exp suicide/OR exp suicide attempt/OR exp suicidal ideation/OR exp self harm/OR exp self-injury/OR exp self mutilation/	168341
2.	suicid*.mp	193432
3.	(Suicid* ADJ3 attempt*).mp	64747
4.	parasuicid*.mp	2292
5.	para-suicid*.mp	87
6.	overdos*.mp	55798
7.	Suicid* AND ideation.mp	29911
8.	(suicid* ADJ3 thought*).mp	6485
9.	selfharm*	108
10.	(self AND harm*).mp	33989
11.	selfinjur*.mp	81
12.	(self AND injur*).mp	69163
13.	selfpoison*.mp	57
14.	self-poison*.mp	4545
15.	(self AND poison*).mp	7958
16.	selfmutilat*.mp	65
17.	self-mutilat*.mp	7358
18.	(self AND mutilat*).mp	7968
19.	self-lacerat*.mp	28
20.	(self AND lacerat*).mp	667
21.	selfcut*.mp	7
22.	(self AND cut*).mp	23891
23.	selfharm*.mp	108
24.	(self AND harm*).mp	33989
25.	selfinjur*.mp	81
26.	(self AND inflict*).mp	6385
27.	self-inflict*.mp	5301
28.	(self destructive AND behavio?r*).mp	5896
29.	(fatal ADJ3 behavio?r*).mp	351
30.	(self AND immolat*).mp	681
31.	self-immolat*.mp.	664
**32.**	**OR(1-31)**	**374883**
33.	exp child/OR exp adolescent/OR exp youth OR exp student/OR exp pupil/	5728114
34.	child*.mp	4956945
35.	(young AND people).mp	125041
36.	(young AND person).mp	23530
37.	youth*.mp	185433
38.	teen*.mp	73759
39.	infant*.mp	979894
40.	junvenile.mp	27
41.	kid.mp	3896
42.	youngster.mp	824
43.	student*.mp	1090005
44.	pupil*.mp	90553
45.	adolescen*.mp	3301925
46.	schoolboy*.mp	1300
47.	(school AND boy*).mp	74913
48.	schoolgirl*.mp	1934
49.	(school AND girl*).mp	74359
50.	schoolchild*.mp	30199
51.	(school AND child*).mp	595660
52.	**OR(33-51)**	8657407
53.	exp School/OR exp Education/OR exp academy/OR exp College/	2045629
54.	school*.mp	1041819
55.	academ*.mp	49059
56.	college.mp	533185
57.	teach*.mp	672906
58.	classroom.mp	86839
59.	(class AND room).mp	5082
60.	(education* ADJ3 setting*).mp	12116
61.	(education* ADJ3 context*).mp	9320
62.	(education* ADJ3 environment*).mp	10706
63.	**OR(53-62)**	3670918
64.	exp qualitative research /OR exp qualitative analysis OR exp qualitative study OR exp qualitative studies	99243
65.	qualitative.mp	454620
66.	interview*.mp	903951
67.	(focus AND group*).mp	202683
68.	ethnograph*.mp	36111
69.	(mixed AND method*).mp	234478
70.	(participant ADJ3 observation).mp	11443
71.	case-stud*.mp	260063
72.	((child* OR adolescen* OR youth OR student OR pupil) ADJ3 (view OR opinion OR experience* OR perception* OR persecptive*).mp	111950
73.	(thematic AND analysis).mp	28864
74.	(content AND analysis).mp	356542
75.	(grounded AND theory).mp	32507
76.	OR(64-76)	2193131
77.	32 AND 52 AND 63 AND 76	5260

## Appendix 2: Data extraction form

**Table:**

**1. Paper ID**	*Title; Publication Type; Publication Status; Author; Year of Publication; Journal Name; Volume Number; Issue no.; Pages; Identified by electronic database?*
**2. Appraiser Information**	*Name of first appraiser; Date first appraisal done DD/MM/YYYY; Name of second appraiser; Date second appraisal done;DD/MM/YYYY;*
**3. Country**	*Country characteristics; Region; City;*
**4. Aim/Research Questions**	
**5. Theoretical Background**	*e.g Phenomenology; Grounded theory; Ethnography; Action research;*
**6. Position of Researcher**	*Reflexivity;*
**7. Methodology**	*e.g. Ethnography, Interview, Focus grou;p*
**8. Context/Setting**	*Educational setting; Academic attainment; SES; Rurality /Urbanity; Gender composition; Ethnic composition;*
**9. Sampling Strategy**	*e.g Purposive; Snowballing;*
**10. Sample Characteristics**	*Sample Size;*
	*Age; Gender; Ethnicity; Professional profile;*
**11. Analysis and Interpretation**	*e.g. Grounded theory; Thematic analysis; Frame analysis;*
**12. Main Findings**	*Outcomes; Themes; Integration with Theory*
**13. Strengths and Limitations**	
**14. Conclusions**	*Policy; Practice; Further Research;*

## Appendix 3: Quality Appraisal Form (Derived from CASP and Campbell et al. (2011))

**Table:**

**1. Paper ID**				
1a. Title				
1b. Publication Type				
1c. Publication Status				
1d. Author				
1e. Year of Publication				
1f. Journal Name				
1g. Volume Number				
1h. Issue no.				
1i. Pages				
1j. Country				
lk. Identified by electronic database?				
1a. Title				
**2. Appraiser Information**				
**2a.** Name of first appraiser				
**2b.** Date first appraisal done DD/MM/YYYY				
**2c.** Name of second appraiser				
**2d.** Date second appraisal done DD/MM/YYYY				
**3. Screening Questions**				
**Questions**	**YES**	**UNCLEAR**	**NO**	**COMMENTS**
**3a.** Does the paper report on the findings from qualitative research and did that work involve both qualitative methods of data collection and data analysis?				
**3b.** Is the research relevant to the synthesis topic?				
**4. Aims**				
**Questions**	**YES**	**UNCLEAR**	**NO**	**COMMENTS**
**4a**. Is there a clear statement of the aims of the research?				
**5. Methodology**				
**Questions**	**YES**	**UNCLEAR**	**NO**	**COMMENTS**
**5a.** Was the research design appropriate to address the aims of the research?				
**6. Theoretical Perspective**				
**Questions**	**YES**	**UNCLEAR**	**NO**	**COMMENTS**
**6a.** Is a theoretical perspective identified?				
**6b.** If yes, which theoretical perspective is identified by the authors?				
1 = Phenomenology				
2 = Grounded Theory				
3 = Ethnography				
4 = Action Research				
5 = Not Classifiable According to Grid				
**6c.** How would you categorise the theoretical perspective?				
1 = Phenomenology				
2 = Grounded Theory				
3 = Ethnography				
4 = Action Research				
5 = Not Classifiable According to Grid				
**7. Sampling**				
**Questions**	**YES**	**UNCLEAR**	**NO**	**COMMENTS**
**7a.** Is it clear which setting the sample was selected from?				
**7b.** Is it clear why this setting was chosen?				
**7c.** Is clear and adequate information given on who was selected?				
**7d.** Was the recruitment strategy appropriate to the aims of the research?				
**7e.** Is the sample size justified by the authors?				
**7f.** Is it clear how many people accepted or refused to take part in the research?				
**7g.** Is it clear why some participants chose not to take part?				
**7h.** Overall, do you consider the sampling strategy appropriate to address the aims?				
**8. Data Collection**				
**Questions**	**YES**	**UNCLEAR**	**NO**	**COMMENTS**
**8a.** Is it clear where the setting of the data collection was?				
**8b.** Is it clear why that setting was chosen?				
**8c.** Is clear how the purpose of the research was explained and presented to the participants?				
**8d.** Is it clear how the data were collected and why?				
**8e.** Is it clear how the data were recorded?				
**8f.** Is there evidence of flexibility or an iterative process in the way the research was conducted?				
**8g.** Is it clear who collected the data				
**8h.** Overall, do you consider the data were collected in a way that addresses the research aims?				
**9. Ethics**				
**Questions**	**YES**	**UNCLEAR**	**NO**	**COMMENTS**
**9a.** Are there sufficient details of how the research was explained to participants for the reader to assess whether ethical standards were maintained?				
**9b.** Does the researcher discuss ethical issues raised by the study?				
**9c.** Was approval sought from the ethics committee?				
**10. Data Analysis**				
**Questions**	**YES**	**UNCLEAR**	**NO**	**COMMENTS**
**10a.** Is it clear how the analysis was done?				
**10b.** Is it clear how the categories/themes were derived from the data?				
**10c.** Is there adequate description of the analysis?				
**10d.** Have attempts been made to feed results back to respondents?				
**10e.** Have different sources of data about the same issue been compared where appropriate (triangulation)?				
**10f.** Was the analysis repeated by more than one researcher to ensure reliability?				
**10g.** Overall, do you consider that the data analysis was sufficiently rigorous to address the aims?				
**11. Research Partnership Relations**				
**Questions**	**YES**	**UNCLEAR**	**NO**	**COMMENTS**
**11a.** Is it clear whether the researchers critically examined their own role, potential bias and influence?				
**11b.** Has the relationship between researchers and participants been adequately considered?				
**11d.** Have attempts been made to feed results back to respondents?				
**12. Findings**				
**Questions**	**YES**	**UNCLEAR**	**NO**	**COMMENTS**
**12a.** Were the findings explicit and easy to understand?				
**12b.** Are the findings discussed in relation to the original research question?				
**13. Justification of Data Interpretation**				
**Questions**	**YES**	**UNCLEAR**	**NO**	**COMMENTS**
**13a.** Are sufficient data presented to support the descriptive findings?				
**13b.** Are quotes numbered/identified?				
**13c.** Do the researchers explain how the data presented in the paper were selected from the original sample?				
**13d.** Do the researchers indicate how they developed their conceptual interpretations of what the data contain?				
**13e.** Are negative, unusual or contradictory cases presented?				
**13f.** Is there adequate discussion of the evidence both for and against the researchers’ interpretations?				
**13g.** Overall, are you confident that all the data were taken into account?				
**14. Transferability**				
**Questions**	**YES**	**UNCLEAR**	**NO**	**COMMENTS**
**14a.** Is there descriptive, conceptual or theoretical congruence between this and other work?				
**14b.** Are the findings of this study transferable to a wider population?				
**15. Relevance and Usefulness**				
**Questions**	**YES**	**UNCLEAR**	**NO**	**COMMENTS**
**15a.** Does the study discuss its contribution to existing knowledge or theory in the field?				
**15b.** How important are these findings to practice?				
**16b.** Does the study consider new areas where research is necessary?				
**16c.** Do they consider the findings in relation to current practice or policy?				
**16d.** Have the researchers discussed whether or how the findings can be transferred to other populations or consider other ways the research may be used?				
**16. Overall Assessment of Study**				
**Questions**	**COMMENTS**			
**16a.** What is your overall view of this study?				
1 = Excellent				
2 = Very Good				
3 = Good				
4 = Not Very Good				
5 = Poor				
6 = Very Poor				
